# Gene rearrangements in the mitochondrial genome of robust tonguefish, *Cynoglossus robustus* (Pleuronectiformes: Cynoglossidae) and a comparative analysis with other *Cynoglossus* fishes

**DOI:** 10.1080/23802359.2019.1637297

**Published:** 2019-12-23

**Authors:** Ha Yeun Song, Jin-Koo Kim, Seonmi Jo, Seung-Hyun Jung, Dae-Sung Lee, Bora Kim, Young Ji Choi, Jong Su Yoo

**Affiliations:** a Department of Genetic Resources Research, National Marine Biodiversity Institute of Korea, Seocheon-gun, Republic of Korea;; b Department of Marine Biology, Pukyong National University, Busan, Republic of Korea

**Keywords:** Mitochondrial genome, Pleuronectiformes, Cynoglossidae, *Cynoglossus robustus*

## Abstract

The complete mitochondrial genome was determined for the Robust tonguefish *Cynoglossus robustus* belonging to the family Cynoglossidae. The length of the complete mitochondrial genome is 16,720 bp, consisting of 13 protein-coding genes, 22 tRNA genes, two rRNA genes, and a control region. Rearrangements of the tRNA^Gln^ and a control region gene were found and tRNA^Gln^ is translocated from the light to the heavy strand. Phylogenetic analysis using mitochondrial genomes of 12 species showed that *C. robustus* formed a well-supported monophyletic group with other *Cynoglossus* species.

The Robust tonguefish *Cynoglossus robustus* (Pleuronectiformes: Cynoglossidae) is commonly found on sandy or muddy bottoms in the China, Korea, and Japan (Yamada et al. 1995). In previous studies, rearrangement related to one tRNA gene and control region were found in *Cynoglossus* species (Kong et al. 2009; Shi et al. 2015; Wei et al. 2016; Bo et al. 2017; Chen et al. 2019). In this study, we first determined the complete mitochondrial genome of *C. robustus* and performed comparative mitogenomic and phylogenetic analysis relationship of this species with other *Cynoglossus* species.

The *C. robustus* specimen was collected from Busan-si, Republic of Korea (34.41N, 129.8E). Total genomic DNA was extracted from tissue of the specimen, which has been deposited in the Marine Fish Resources Bank of Korea (MFRBK) (Voucher No. PKU58916). The mitogenome was sequenced and assembled using Illumina Hiseq 4000 sequencing platform (Illumina, San Diego, CA) and *SOAP denovo* assembler at Macrogen Inc. (Korea), respectively. The complete mitochondrial genome was annotated using MacClade ver. 4.08 (Maddison and Maddison, 2005) and tRNAscan-SE 2.0 (Lowe and Chan 2016).

The complete mitochondrial genome of *C. robustus* (GenBank accession no. LC482305) is 16,720 bp long and includes 13 protein-coding genes, 22 tRNA genes, two rRNA genes, and a control region. The overall base composition is 30.32% A, 24.95% C, 15.15% G, and 29.58% T, with a bias on AT content (60.8%). Similar to the mitogenomes of other vertebrates, the AT content is higher than the GC content (Saccone et al. 1999). The 12S rRNA (943 bp) and 16S rRNA genes (1685 bp) are located between tRNA^Phe^ and tRNA^Val^ and between tRNA^Val^ and tRNA^Leu(UUR)^, respectively. Of the 13 protein-coding genes, 11 begin with an ATG start codon; the exception being the *COI* gene and *ND3*, which start with GTG and ATT, respectively. The stop codon of the protein-coding genes is TAA in *ND1, COI*, *ATP8*, *ND4L, ND5*, *ND6* and *Cytb*; TAG in *ND2*; TA in *ATP6* and *COIII*; and T– – in the remaining three genes.

We detected gene rearrangements of mitogenome in the *C. robustus*. The tRNA-Gln gene encoded by the light strand has translocated to the heavy strand and the control region translocated downstream to the place between ND1 and tRNA-Ile genes. Additionally, unique character of this mitogenome, gene order of CR-Ile-Gln-Met, was found through comparison of the mitogenome with other *Cynoglossus* species, forming the gene order of CR-Gln-Ile-Met (Kong et al. 2009; Shi et al. 2015; Wei et al. 2016; Bo et al. 2017; Chen et al. 2019).

Phylogenetic trees were constructed by the maximum-likelihood method using MEGA 7.0 software (Kumar et al. 2016) for the newly sequenced genome and a further 12 mitochondrial genome sequences downloaded from the National Center for Biotechnology Information. We confirmed that *C. robustus* formed a monophyletic group with other *Cynoglossus* species (Figure 1). The novel mitogenome features of the *C. robustus* could contribute to a better understanding the molecular mechanisms of gene rearrangements in *Cynoglossus* species.

**Figure 1.**
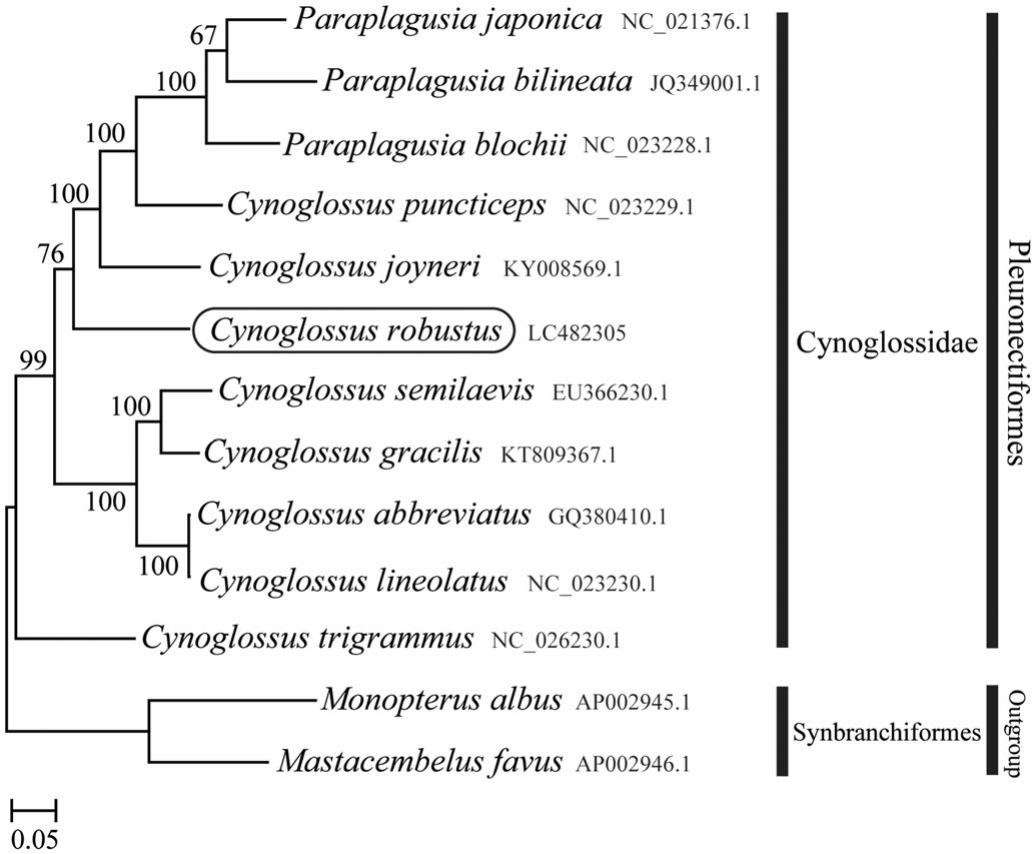
Phylogenetic position of *Cynoglossus robustus* based on a comparison with the complete mitochondrial genome sequences of 13 species. The analysis was performed using MEGA 7.0 software. The accession number for each species is indicated after the scientific name.
